# A detailed spatial analysis on contrasting cancer incidence patterns in thyroid and lung cancer in Toronto women

**DOI:** 10.1186/s12889-016-3634-4

**Published:** 2016-09-08

**Authors:** Patrick Brown, Hedy Jiang, Shereen Ezzat, Anna M. Sawka

**Affiliations:** 1 Prevention and Cancer Control, Cancer Care Ontario, 620 University Avenue, Toronto, Ontario M5G 2L7 Canada; 2 Endocrine Oncology, Princess Margaret Cancer Centre, 585 University Avenue, 9NU-986, Toronto, Ontario M5G 2N2 Canada; 3 Division of Endocrinology, Department of Medicine, University Health Network and University of Toronto, Toronto General Hospital, 200 Elizabeth Street, 12 EN-212, Toronto, Ontario M5G 2C4 Canada

**Keywords:** Geographic spatial analysis, Small area disease mapping, Incidence rate, Thyroid cancer, Lung cancer, Epidemiology, Female

## Abstract

**Background:**

Thyroid cancer has been rapidly rising in incidence in Canada; however, in contrast, lung cancer appears to be decreasing in incidence in Canadian men and stable in women. Moreover, disease-related mortality risk is generally very low in TC but high in LC. We performed a geographic spatial analysis in metropolitan Toronto, Canada to determine if there is regional variability of respective risks of thyroid cancer (TC) and lung cancer (LC), among women. Women were of particular interest for this study, given their known predilection for thyroid cancer.

**Methods:**

The postal codes of all females with TC or LC, residing in metropolitan Toronto from 2004 to 2008, were geocoded to point locations according to 2006 Canadian Census data. The data were analysed using a log-Gaussian Cox Process, where the intensity of age-adjusted cancer cases was modelled as a log-linear combination of the population at risk, explanatory variables (race, immigration, and median household income), and a residual spatially varying random effect. For each respective malignancy, statistical models were fit to make quantify the relationship between cancer incidence and explanatory variables.

**Results:**

We included 2230 women with TC and 2412 with LC. The distribution of TC and LC cases contrasted inversely among Toronto neighbourhoods with the highest TC incidence in the Northeast and the highest LC incidence in the Southeast. A higher proportion of Asian ethnicity was associated with higher regional risk of TC and lower risk of LC. A higher proportion of recent immigrants was associated with increased LC and lower TC risk, whereas median household income and proportions of African ethnicity were not significantly associated with risk of either cancer, after adjustment for other socio-demographic variables.

**Conclusions:**

We observed contrasting regional distributions of female TC and LC cases in Toronto. The differences were partly attributed to ethnic composition variability and the proportion of recent immigrants, but substantial unexplained residual variation of incidence patterns of these malignancies exists, suggesting that more individual-level research is needed to explain the regional variability of incidence of these malignancies.

## Background

The number of individuals diagnosed annually with thyroid cancer (TC) is approximately 289,000 globally [1], including approximately 6,300 Canadians [2], 62,450 Americans [3]. Furthermore, TC accounts for the most rapid rise in incidence of any cancer in Canada and the United States [2, 3]. TC carries one of the lowest risks of disease-related mortality among malignancies [2, 3]. Approximately three out of every four TC diagnoses are made in women, reflecting a marked female sex predilection [3]. In Canada, there is significant geographic variability in TC incidence rates across the nation [2, 4], with particularly elevated rates observed in urban centers [5], and especially the province of Ontario [2]. In contrast to many other malignancies, smoking is associated with a reduced risk of TC [6]. An in-depth analysis of regional distribution of Canadian TC cases in population-dense urban centers, including representation of a variety of racial and immigrant groups across a spectrum of socioeconomic class, is of great interest, in unraveling current incidence trends of this malignancy.

There were approximately 26,600 Canadians [2] and 221,200 Americans [3] diagnosed with lung cancer (LC) in 2015. In Canada, lung cancer (LC) is currently the second most common malignancy diagnosed in women and the third most common in men, with a slightly lower age- standardized incidence rate in women (48 per 100,000 population) compared to men (58 per 100,000 population) [2]. LC carries the highest cancer-related death of all cancers in Canada [2] and the United States [3]. LC incidence has been decreasing in men and has been relatively stable in Canadian women since 2006, with future projected decreases in women attributable to tobacco control, given that smoking is a well-established risk factor for this malignancy [2]. There is significant regional variability of LC incidence rates across Canadian provinces [2]; the highest age-standardized incidence rates of LC are estimated in Quebec, and the risk of LC is more than 40 % higher in both sexes in that province compared to the neighboring Ontario [2]. Taken together, these data are suggestive of contrasting epidemiologic risk factors and disease behavior of TC and LC. As such, in this work, we examined the regional variability of respective TC and LC incidence among women in Toronto neighborhoods and explored the relationship of incidence patterns with socio-demographic variables (ethnicity, immigration, and household income). Metropolitan Toronto is Canada’s most populated urban center rendering it an ideal setting to examine disease-ethnic associations. This study was focused on women, given their known predilection to TC risk [3]; whereas in contrast, the risk of LC may be slightly lower in women compared to men [2]. Restricting our analysis to the female gender would also influence any potential impact of gender difference on the interpretation of results. The public health implications of our study are that it provides some insight into the regional incidence patterns, potential explanatory factors, and health resource utilization implications of these malignancies for the region of interest.

## Methods

### Data

Our population of interest was women living within the boundaries of Metropolitan Toronto, Canada, during the 2006 Census of Canada. We included women from Toronto who had been diagnosed with TC or LC within the years of 2004 to 2008. A valid residential postal code (traced to our Postal Code Conversion File), for regions reporting on race and household income in the 2006 census, were required for cases to be included. The rationale for restricting the analysis to females is that females are at much higher risk of thyroid cancer than men [3]. Publicly available data from the 2006 Census of Canada were used for the explanatory variables in the model, which were log-transformed median yearly log-transformed household income (in Canadian dollars), Ethnic ancestry (race), recent immigration (within 5 years prior to the 2006 census), and the female population by 5-year age group. The geographic units for the population and explanatory variables were the 3557 Dissemination Areas (or DA’s), which are defined by Statistics Canada as a small areas typically including 400 to 700 individuals [7]. The proportion of individuals of African ancestry was defined as those having ‘African origins’ or ‘Caribbean origins’, or ‘Arab origins’, whereas the proportion of individuals of Asian ancestry were defined as those having ‘West Asian origins’, ‘South Asian origins’, or ‘East and Southeast Asian origins.’ The population density was assumed to be homogenous within respective DA’s, as were the potential explanatory variables (income, race, and immigration).

The six-digit postal codes of all cases of female TC (ICD-9 Diagnosis Code 193 and ICD-10-CM Diagnosis Code C73) and LC (ICD-9 Diagnosis Code 162 and ICD-10-CM Diagnosis Code C34.9) in Toronto from 2004 to 2008 (5 years centered on the 2006 census) were obtained from the Ontario Cancer Registry [8]. The Ontario Cancer registry includes data on all newly diagnosed malignancies (except for basal and squamous cell skin cancer), diagnosed in the province [8]. The major data sources utilized for the definition of cases by the Ontario Cancer Registry include; pathology reports from hospitals and community laboratories, hospital discharge and day surgery records from the Canadian Institute for Health Information, consultation and treatment reports from Regional Cancer Centres, and death certificates from the Ontario Registrar General [8]. Records from the multiple data sources are consolidated into one or more primary cases of cancer. A resolved record is then generated representing lung and thyroid cases. The postal codes of residence of all cases were then geocoded to point locations using the 2006 Postal Code Conversion File. This study was approved by the University Health Network Research Ethics Board (Study identifier 15-8758-CE) and Cancer Care Ontario.

### Availability of data and materials

As the data used in this study are comprised of personal health information, the data cannot be shared for reasons of privacy and confidentiality (per Cancer Care Ontario policy).

### Consent for publication

Individual informed consent was not required for this registry-based study. This manuscript does not describe any individual person’s data in any form so a consent to publish is not applicable for this study.

### Statistical analysis

The locations of TC and LC cases were modelled as a spatial point process, specifically a log-Gaussian Cox Process [9], where the intensity of cancer cases was determined from a combination of the population at risk, explanatory variables, and spatially varying ‘residual’ random effect. For each cancer type, a ‘baseline’ spatial intensity surface was created from the census population data and rates by 5-year age groups for all Canada, during the 2004 to 2008 year period. This calculation produced an expected count for each DA, by summing the product of rates and populations over age groups, which is converted to an expected count per square kilometer (km) by dividing each DA’s surface area. Denoting this baseline intensity by E(s) and a relative risk surface by λ(s), case locations were modelled as a Poisson point process with an intensity function ρ(s) = E(s) λ(s) being the product of these two terms. Substantial numbers of cases would be expected at locations s where ρ(s) is large, and an excess of cancer risk at s occurs when ρ(s) > E(s) and hence λ(s) >1.

The relative risk *λ*(*s*) was defined as a log-linear combination of spatial explanatory variables *X*(*s*), a vector of the logged median household income, proportion of individuals of Asian or African ethnicity, and the proportion of recent immigrants at s. Additionally, a residual random term *U*(*s*) describes variation in risk not accounted for by the explanatory variables. More formally, the model is defined as log[*λ*(*s*)] = *X*(*s*)*β* + *U*(*s*), with *β* being a set of regression coefficients (or log relative risk parameters) and *U*(*s*) is a Gaussian random field with standard deviation *σ* and a Matern spatial correlation function having range parameter *φ* and shape parameter 1.

Bayesian inference with an integrated nested Laplace approximation [10] algorithm was used for model fitting, using the geostatsp package [11] of the R programming language [12]. Flat prior distributions were used for the *β* parameters, and reasonably uninformative prior distributions for *φ* and *σ* had 95 % prior intervals of (300 m, 5 km) and (0.05, 1) respectively. The continuous *U*(*s*) surface was approximated by a gridded (or raster) surface with 170 m by 170 m cells covering the study region. Separate models were fit to TC and LC case data.

## Results

### TC and LC cases included in the analysis

We identified 2501 incident TC and 2910 incident LC cases among Toronto women during the period of study. However, the following numbers of TC and LC cases were excluded for the following reasons: residential postal codes did not match the Postal Code Conversion File (15 TC, 27 LC), and residential postal codes were in census subdivisions without household income data (256 TC, 471 LC). Thus, the final number of included cases was 2230 women diagnosed with TC and 2412 diagnosed with LC.

### Description of the census DA’s in Toronto

**Fig. 1:**
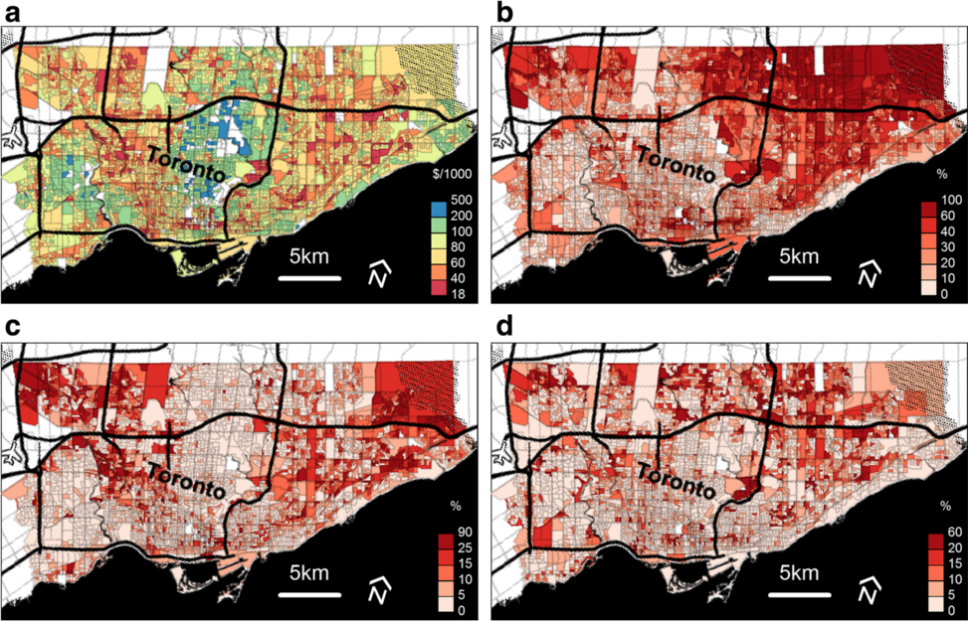
Epidemiologic characteristics of census Dissemination Areas in Toronto (Background © Stamen Design). Legend: **a**. Median household income (log-transformed). **b**. Proportion of Asian ancestry. **c**. Proportion of African ancestry. **d**. Proportion of recent immigrants (within 5 years prior to the 2006 census)

Among the 3577 census dissemination areas identified in Toronto, the proportion of individuals of Asian race was a median of 0.26 (inter-quartile range, 0.13, 0.47) and the proportion of individuals of African race was 0.05 (inter-quartile range, 0.02, 0.12). The log-transformed median yearly household income was $69,139 Canadian (inter-quartile range, $52,328, $88,647). The proportion of recent immigrants was a median of 0.05 (inter-quartile range, 0.02, 0.11). The spatial distribution of ethnicity (Asian, African), median household income, and recent immigrants among Toronto DA’s are shown in Fig. 1. Some patterns that were observed included that individuals of Asian and African descent were more likely to reside in the Northeast and Northwest areas of Toronto. Furthermore, the Northeast and Central-north areas were among the regions with the highest median family income.

### Regional distribution of TC and LC cases and epidemiologic associations

**Fig. 2:**
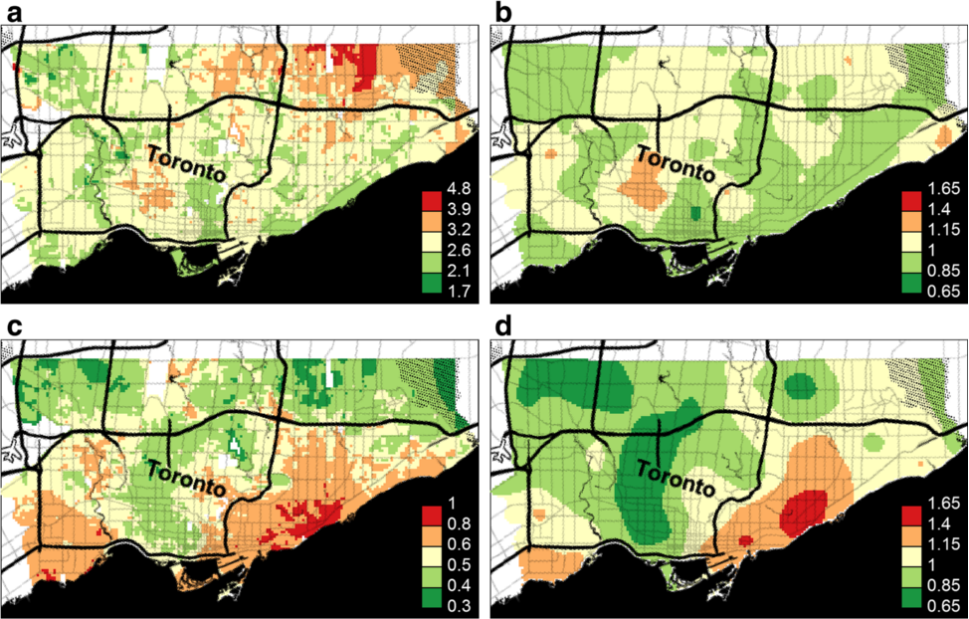
Predicted (or posterior median) relative risk λ(s) and residual spatial variation exp [U(s)] for Lung and Thyroid cancers amongst women in Toronto (Background ©Stamen Design). Legend: **a**. Thyroid cancer relative risk. **b**. Thyroid cancer residual variation. **c**. Lung cancer relative risk. **d**. Lung cancer residual variation

**Fig. 3:**
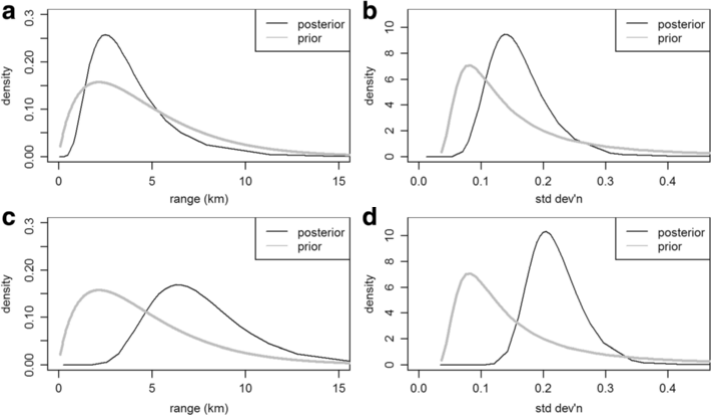
Prior and posterior distributions for selected model parameters. Legend: **a**. Thyroid cancer range *ϕ.* **b**. Thyroid cancer standard deviation *δ.* **c**. Lung cancer range *ϕ.* **d**. Lung cancer standard deviation *δ*

Upon examining the regional distribution of female TC and LC cases, we observed a relatively higher density of TC cases in the Northeast part of the city (Fig. 2a), and a higher density of LC cases in the Southeast (Fig. 2c). Lung cancer risk in Toronto is lower than the Canadian average and thyroid cancer is more common in Toronto than elsewhere in Canada, which is reflected in relative risk for LC being below 1.1 and relative risk for TC being above 1.5 throughout the city. From the map of residual spatial random effect for LC (Fig. 2d), a strong spatial effect is observed in the Central Southeast. However, the map of TC cases does not show the same pattern (Fig. 2b). These data strongly suggest that the risks for TC and LC are highly variable regionally, but that the risk for each of these malignancies appears to be independent. Table 1 shows parameter estimates for both models along with 95 % posterior credible intervals, and the larger range parameter for LC of 7.2 km (against 3.3 km for TC) reflects the smoother contour lines for residual LC risk in Fig. 2b. The standard deviation parameter is larger for LC, however, at 0.21 versus 0.15 for TC, which is reflected in the surface in Fig. 2b showing relative risks for TC closer to 1.0 than the comparable values for LC in Fig. 2d. Figure 3 shows prior and posterior distributions for the range and standard deviation parameters, with the larger LC dataset shifting posterior distributions further from their priors than the smaller number of TC cases do.

**Table 1:** Estimates of relative risk (posterior medians) and 95 % posterior credible intervals for risk ratios of inter-quartile ranges, range parameters φ (in km) and standard deviation parameters σ of the residual spatial variation^a^

Variable	Thyroid cancer relative risk estimate (0.025 qt, 0.975qt)^a^	Lung cancer relative risk estimate (0.025 qt, 0.975qt)^a^
Median household income (Log-transformed, Canadian dollars)	0.97 (0.89, 1.06)	0.94 (0.86, 1.02)
Asian ancestry	1.21 (1.10, 1.33)	0.80 (0.71, 0.90)
African ancestry	0.95 (0.889, 1.01)	0.98 (0.91, 1.04)
Recent immigrant	0.90 (0.84, 0.98)	1.09 (1.01, 1.18)
Range (km)	3.31 (1.142, 9.30)	7.20 (3.63, 14.25)
Standard Deviation	0.15 (0.09, 0.28)	0.21 (0.15, 0.32)

Legend: ^a^The parameters in Table 1 for yearly household income, Asian ancestry, African ancestry, and recent immigrants (ie. immigrants within the prior 5 years) show estimates and 95 % credible intervals for relative risks, or exp(*β*), associated with each of the explanatory variables. These values are risk ratios for inter-quartile ranges, or the ratio between risks for regions in the 75th percentile and the 25th percentile for each variable, on the natural scale with 1.0 indicating no effect

The relative risks and associated 95 % for credibility variables for explanatory variables are shown in Table 1, with effect sizes reported as relative risks for inter-quartile ranges. A significantly increased density of age-adjusted TC cases occurs in areas including proportionally more individuals of Asian ethnicity, as expressed as a mean relative risk [RR] of 1.21 (95 % credibility interval [CRI], 1.10, 1.33), whereas the risk of TC was not significantly impacted in areas including proportionally more individuals of African ethnicity (RR 0.95, 95 % CRI, 0.89, 1.01). The risk of TC in areas with a higher proportion of recent immigrants was slightly reduced, as expressed as a RR of 0.90 (95 % CRI, 0.84, 0.98). The age-adjusted density of TC cases was not significantly associated with regional median household income (mean RR 0.97, 95 % CRI, 0.89, 1.06). In contrast to TC, the age-adjusted LC risk was reduced in areas including proportionally more Asian women (mean RR 0.80, 95 % CRI, 0.71, 0.90). However, regional representation of African ethnicity was not significantly associated with age-adjusted LC risk (mean RR 0.98, 95 % CRI, 0.91, 1.04). Higher regional median household income level was also not significantly associated with age-adjusted risk of LC (mean RR 0.94, 95 % CRI, 0.86, 1.02). The risk of LC in areas including a higher proportion of recent immigrants was slightly increased, as expressed by a mean RR of 1.09 (95 % CRI, 1.01, 1.18).

## Discussion

In comparing the regional distribution of TC and LC cases among Toronto women, we observed that the neighbourhoods where the density of TC was the highest, were not overlapped with the areas where the density of LC cases was the highest. In comparing the association between distribution of ethnic background and cancer incidence, a higher regional density of Asian race was associated with a significantly increased age-adjusted density of TC cases, but significantly reduced relative risk of LC. However, a higher regional density of African race was not significantly associated with the age-density of either TC nor LC female cases. A higher regional density of recent immigrants was associated with a slight reduction in TC relative risk but slight increase in LC relative risk. In a national Canadian study, Carriere et al. reported an inverse gradient of overall cancer incidence rates and regional concentration of foreign-born individuals; however the opposite relationship was observed for thyroid cancer [13]. Similarly, we have previously reported a positive association between proportion of immigrants and TC incidence among health regions within the province of Ontario [14]. It is important to note that in this analysis, we defined immigrant status by recent immigration (within 5 years) and not on the basis of foreign birth without time restriction, which may account for some of the difference in our findings. Interestingly, Horn-Ross et al. reported that in an analysis of California data from the California Cancer Registry, for the period of time similar to our analysis (years 2003 to 2009) the average annual age-adjusted incidence rate for thyroid cancer per 100,000 population was relatively comparable in Non-hispanic Whites (12.03, 95 % confidence interval [CI] 11.75, 12.32) to that of Asian/Pacific Islanders born in the United States (12.40, 95 % CI, 10.91, 14.03), but it appeared slightly lower in foreign-born Asian Pacific Islanders (9.17, 95 % CI, 8.62, 9.76) [15]. Thus, the existing North American reports on any association between immigration and thyroid cancer incidence appear conflicting. Higher socioeconomic status (reflected by regional median household income) was not significantly independently associated TC nor LC age-adjusted risk in this study, after adjustment for race, and immigration in our study. An inverse association between socioeconomic status and LC incidence has been previously reported [16–18], although it appears to be attenuated by adjustment for smoking history [19]. A positive association between socioeconomic status and TC risk has been previously reported in other studies [5, 20–24], but was not observed in our study, which may in part, relate to differences in the method of analysis (spatial analysis adjusted for multiple variables in our study), classification of socioeconomic status, variability of incomes within studied regions, differences in healthcare delivery, gender differences of study populations (as our study was restricted to women), or other lifestyle or cultural factors. In spite of the observed contrasting epidemiologic associations with TC and LC risk in our study, some of the excess regional risk remained unexplained, as reflected by the residual risk plots generated by our analyses.

The incidence of TC in Toronto is known to be higher than that of the majority of other large Canadian cities, after adjustment for demographic and socio-economic variables [25]. Furthermore, Corsten et al. have recently reported that the incidence of TC in the northern Greater Toronto Area (eg. Markham, Vaughn, and Richmond Hill) is significantly higher than in the Toronto city core [25]. Although our method of analysis was different and we restricted our analysis to women, our study results generally corroborate those published recent findings suggesting increased TC relative risk in the northern Greater Toronto area. Individual-level analyses are now required to explore potential environmental, healthcare access, or other potential factors contributing to the disproportionately high risk of TC in the northern Greater Toronto Area.

Another finding of this study, was the observation of the relationships between regional race representation and cancer incidence. Specifically, we observed an inverse independent association of regional representation of Asian ancestry, with risk of TC (increased) and LC (reduced) among Toronto women. Jin et al. have recently analyzed data from the Surveillance, Epidemiology, and End Results [SEER] Program from 2009 to 2011 from eight states, and observed that in Asian American women, the risk of TC was comparable (21.5 per 100,000 population, 95 % confidence interval [CI] 20.8–22.2) to that of Non-hispanic white women (22.4, 95 % CI 22.1–22.7), whereas the risk of LC was reduced in Asian women (28.6, 95 % CI 27.8, 29.5) compared to Non-Hispanic white women (59.4, 59.0, 59.8) [26]. With respect to the risk of papillary thyroid cancer (the most common type of TC), Aschebrook-Kilfoy et al., has previously reported that among American women in the SEER database, the risk of papillary thyroid cancer was greatest among Asian Americans and lowest among African Americans [27]. Furthermore, Aschebrook-Kilfoy observed that among Asian Americans, regional papillary thyroid cancer incidence rates were highly variable, ranging from 5.3 per 100,000 population in the state of Connecticut to 9.5 per 100,000 in Iowa [27]. Yet we did not observe any significant association of African race and TC nor LC risk in our study. Previous reports analyzing data from SEER, have suggested a reduced risk of TC in African American women compared to white women [27, 28]. Our study highlights the importance of advancing our understanding of the relationship between race (and the genetics of race), the environment, lifestyle, and cancer susceptibility.

Some of the strengths of our study included the retrieval of epidemiologic and cancer incidence data from well-established databases. Furthermore, the number of LC and TC cases included in this study was relatively large. Another strength of this study was the use of Bayesian inference with the INLA package in the R statistical programming language; the INLA software has made fitting Geostatistical models to count data and point location data a practical and convenient option for studies of this kind, in contrast to earlier inference methodologies which were computationally intensive and required specialist knowledge (see the Discussion in Diggle et al. [29]). Some limitations of this study include the lack of details of individual data (such as individual smoking status, personal income, or race/ethnicity, or generational status), the relatively limited geographic scope of the analysis, the restriction of the analysis to women, and the lack of specific details relating to the cases of malignancy (eg. how diagnosed, disease stage, treatments, and outcomes). Also, it is possible that regional level variables such as ethnicity may be capturing other associated variables from neighbourhoods, rather than reflecting ethnicity itself. Furthermore, more spatially related factors could have been included to attempt to explain the spatial variation observed in the study, but our analyses were limited to some extent by resource and time limitations, as well as the availability of well-validated relevant potentially explanatory data.

## Conclusion

In conclusion, our main finding was a remarkably contrasting incidence pattern for thyroid and lung cancer in Toronto women. This research highlights how contrasting spatial variation of incidence rates of different malignancies (such as thyroid and lung), may be relevant to explore in understanding the underlying mechanisms of susceptibility and causative factors associated with their incidence. Furthermore, the spatial analyses presented herein exemplify a cost-efficient means to account for important confounders in conducting exploratory causation research for human malignancies. In future research, information on social, genetic, and reporting effects should be taken into account in detailed spatial and individual-level analyses, to better inform our understanding of the incidence patterns and possible causative or preventative factors, in the development of thyroid and lung malignancies.

## Abbreviations

95 % CRI: 95 % credibility interval
DA: Dissemination area
LC: Lung cancer
RR: Relative risk
TC: Thyroid cancer
